# Effect of Surface Coverage of Gold Nanoparticles on the Refractive Index Sensitivity in Fiber-Optic Nanoplasmonic Sensing

**DOI:** 10.3390/s18061759

**Published:** 2018-05-31

**Authors:** Wei-Te Wu, Chien-Hsing Chen, Chang-Yue Chiang, Lai-Kwan Chau

**Affiliations:** 1 Department of Biomechatronics Engineering, National Pingtung University of Science and Technology, Pingtung 912, Taiwan; 2 Department of Chemistry and Biochemistry and Center for Nano Bio-Detection, National Chung Cheng University, Chiayi 621, Taiwan; saesozj@yahoo.com.tw (C.-H.C.); standford_chiang@hotmail.com (C.-Y.C.)

**Keywords:** particles plasmon resonance, extinction spectrum, gold nanoparticle

## Abstract

A simple theoretical model was developed to analyze the extinction spectrum of gold nanoparticles (AuNPs) on the fiber core and glass surfaces in order to aid the determination of the surface coverage and surface distribution of the AuNPs on the fiber core surface for sensitivity optimization of the fiber optic particle plasmon resonance (FOPPR) sensor. The extinction spectrum of AuNPs comprises of the interband absorption of AuNPs, non-interacting plasmon resonance (PR) band due to isolated AuNPs, and coupled PR band of interacting AuNPs. When the surface coverage is smaller than 12.2%, the plasmon coupling effect can almost be ignored. This method is also applied to understand the refractive index sensitivity of the FOPPR sensor with respect to the non-interacting PR band and the coupled PR band. In terms of wavelength sensitivity at a surface coverage of 18.6%, the refractive index sensitivity of the coupled PR band (205.5 nm/RIU) is greater than that of the non-interacting PR band (349.1 nm/RIU). In terms of extinction sensitivity, refractive index sensitivity of the coupled PR band (−3.86/RIU) is similar to that of the non-interacting PR band (−3.93/RIU). Both maximum wavelength and extinction sensitivities were found at a surface coverage of 15.2%.

## 1. Introduction

Gold nanoparticles (AuNP) have been used over a wide range of fields in chemical and biochemical analyses such as clinical analysis [1,2,3,4,5,6], molecular diagnostics [7,8] bio-interaction analysis and drug discovery [9], agricultural diagnostics [10,11], environmental monitoring [12], and food safety analysis [13,14,15]. The energy density of an AuNP varies with particle size [16] and shape [17,18], leading to a special absorption band of AuNP known as the plasmon resonance (PR) band [19]. This absorption band results when the frequency of an incident light is resonant with the collective dipole oscillation of the conduction electron in the AuNP and is known as particle plasmon resonance (PPR), also known as localized surface plasmon resonance (LSPR). This feature was first explained by Faraday [20], until 1908, Mie adopted Maxwell’s equation using a single spherical particle for description of the extinction spectrum [21]. The absorption peak of particle plasmon resonance is very sensitive to the change of the surrounding medium, and thus especially suitable for the development of optical sensors.

In recent years, various forms of assays based on AuNPs have been developed, such as solution-phase-based [4,12,14], slide-based [3,11,15,22], and optical fiber-based [1,2,5,6,10,23,24,25,26,27,28,29,30]. Among the PPR-based assays, optical fiber-based PPR sensors have shown many advantages such as high sensitivity, wide linear dynamic range, normalized sensor response, being easy-to-operate, ease-of-miniaturization, simple optical design, and inexpensive sensor fabrication processes. It has been suggested that the excitation of guided modes in total internal reflection can drastically increase light/matter interaction leading to large amplification of the sensitivity in biosensing measurements [31]. Since the size, shape, surface coverage, and even surface distribution of AuNPs have been considered as major factors in characterizing the optical properties of an AuNPs-coated surface, extensive research has been undertaken to optimize such factors to enhance the sensitivity, reproducibility, and robustness of this type of sensors [32,33,34,35,36,37]. As there are very limited number of reports in exploring the effects of surface coverage and surface distribution of AuNPs on the sensitivity of PPR sensors [33,34,36], this research aims at investigating such factors.

The optical fiber-based PPR sensor, named fiber optic particle plasmon resonance (FOPPR) sensor, is based on immobilization of AuNPs on the unclad section of an optical fiber to form a sensing fiber [23,25]. As the quality of the exposed fiber core surface and the coverage of AuNPs on the fiber core surface will influence the sensitivity of the FOPPR sensor, fiber surface quality analysis has been discussed in literature [38]. However, the effects of surface coverage and surface distribution of AuNPs on the sensitivity of PPR sensors are not trivial since direct tools to measure the surface coverage and surface distribution of AuNPs on the fiber core surface are not available. The size and size distribution of AuNPs in solution can be measured by transmission electron microscopy (TEM) [39], dynamic light scattering (DLS) [40] and UV-vis spectroscopy [41], and the surface coverage of AuNPs on the fiber core surface can be measured and analyzed by scanning electron microscopy (SEM). However, SEM cannot quickly and instantly monitor the surface coverage and surface distribution of AuNPs on the fiber core and glass surface. In addition, SEM measurements need to coat a metal film layer on the fiber core surface, which is destructive. As such, obtaining a macroscopic picture about the surface coverage and surface distribution of AuNPs on the fiber core surface in a short time and non-destructively are certainly helpful for the quality assurance of the sensing fibers during production.

Although measurement of an evanescent field extinction spectrum of a sensing fiber can be achieved [23], the spectral features are complicated by the number of total internal reflections in the fiber and the penetration depth of the evanescent wave, which are all related to the angle of the incident light. To correctly measure and analyze the extinction spectrum of the sensing fiber, the incident light should satisfy all propagation modes in the fiber. Furthermore, Mie theory [21] is only suitable for analyzing the extinction spectrum of a single spherical particle. In reality, the surface distribution of AuNPs is random. Hence, coupling of plasmon modes between two AuNPs may happen, resulting in a red-shifted plasmon resonance (PR) band. As a result, the extinction spectrum may consist of non-interacting and interacting PR bands. Therefore, a simple theoretical model to analyze the extinction spectrum of AuNPs on the fiber core and glass surface and a fiber optic extinction spectrometry measurement system are proposed. By this approach, the surface coverage and surface distribution of the AuNPs on the fiber core surface were analyzed and the refractive index sensing experiments were carried out by using this spectral measurement system to characterize the effects of surface coverage and surface distribution of AuNPs on the sensitivity of the FOPPR sensor.

## 2. Materials and Methods

### 2.1. Theoretical Model

**Figure 1:**
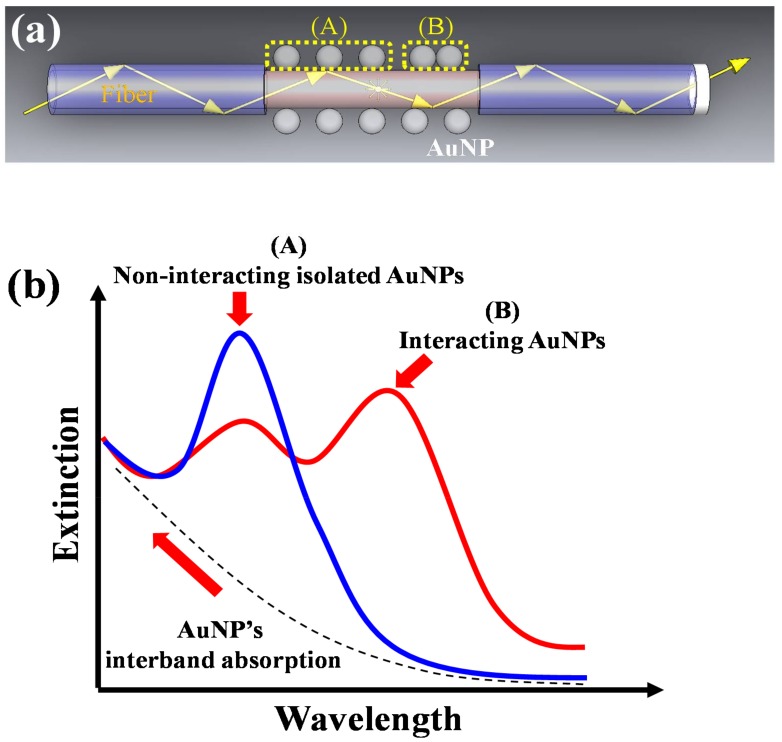
(**a**) Types of distribution of AuNPs on fiber core surface; (**b**) Illustration of the fiber optic extinction spectra with two types of AuNP distributions.

For a single spherical gold nanoparticle which is small compared to the wavelength *λ* of the exciting light, only the dipole absorption effect is assumed to contribute significantly to the extinction cross section (*C_ext_*) and thus the Mie theory reduces to the following relationship [16,21,42,43]:

(1) $$C_{ext}=\frac{24\pi^{2}R^{3}\varepsilon_{m}^{3/2}}{\lambda }\frac{\varepsilon_{2}(\lambda ,R)}{\left[{\left(\varepsilon_{1}(\lambda ,R)+2\varepsilon_{m}\right)}^{2}+\varepsilon_{2}^{2}(\lambda ,R)\right]}$$

where *ε*_1_(*λ*, *R*) is the real part of the dielectric function of the spherical metal nanoparticle; *ε*_2_(*λ*, *R*) is the imaginary part of the dielectric function of the spherical metal nanoparticle, *R* is the radius of the spherical metal nanoparticle; *λ* is the wavelength of the incident light; *ε_m_* is the dielectric constant of the external environment, which can be expressed as *ε_m_* = *n_m_*^2^; *n_m_* is the refractive index of the external environment. The PR absorption band of AuNPs with size of ~3–25 nm are size independent [16] and is intraband in nature [44]. However, AuNPs smaller than ~2 nm exhibit a featureless straight line stretching from the near infrared through near-ultraviolet and is attributed to the 5d to 6sp interband absorption [45], as illustrated by the dashed-line in Figure 1b. The absorbance *A_λ_* of the interband transition at wavelength *λ* falls exponentially with *λ* and the absorption curve can be represented by [44,46]:

(2) $$A_{\lambda }=c_{1}\exp(-c_{2}\lambda )$$

where *c*_1_ and *c*_2_ are constants. The interband absorption also applies to AuNPs larger than ~2 nm [46] and thus AuNPs larger than ~2 nm have absorption spectral features due to both interband and intraband transitions.

For a FOPPR sensor, there are many AuNPs on the core surface of the unclad section of the optical fiber, as shown in Figure 1a. When the AuNPs on the surface behave as non-interacting isolated nanoparticles (i.e., the gap between two AuNPs is at least 2.5 times the nanoparticle size [47,48]), as illustrated by region (B) of Figure 1a, then the polarization field due to the surrounding nanoparticles is negligible and a single intraband absorption peak results. The width of the extinction peak has been suggested to be a Lorentzian line shape in the frequency domain and the absorption curve can be expressed as [49]:

(3) $$A_{\lambda }=A_{0}+\frac{2c_{3}}{\pi }\frac{w}{4{\left(\lambda -\lambda_{R}\right)}^{2}+w^{2}}$$

where *A_λ_* is the absorbance of the intraband transition at wavelength *λ*, *A*_0_ is the baseline value; *λ_R_* is the peak wavelength of the non-interacting PR band; *w* is the full-width at half-maximum (FWHM); *c*_3_ is a constant. Including the interband absorption, the extinction spectrum in this case is shown by Curve A of Figure 1b.

When the AuNPs get very close, individual plasmon oscillations on neighboring nanoparticles can couple via their near-field interaction, resulting in coupled plasmon modes [50]. In practice, during the self-assembly of AuNPs on surface, the distance between any two nanoparticles varies from pair to pair. Hence, the absorption peak of the coupled plasmon modes is likely a superposition of many PR bands corresponding to a distribution of nanoparticle pairs with various separation distances [51] and can be approximated as a Gaussian band [44,46,51]. The absorption curve of the coupled PR band can be expressed as:

(4) $$A_{\lambda }=c_{4}\exp[-(\lambda -\lambda_{2rd})/2w_{2rd}{}^{2}]$$

where *λ*_2*rd*_ is the peak wavelength of the coupled PR band; *w*_2*rd*_ is the FWHM of the coupled PR band; *c*_4_ is a constant. Thus, the overall extinction spectrum can be approximated by a combination of bands, including the interband absorption, the non-interacting PR band due to isolated AuNPs, and the coupled PR band of interacting AuNPs, as shown by Curve B of Figure 1b.

### 2.2. Experimental Section

#### 2.2.1. Preparation of Sensing Fibers

**Figure 2:**
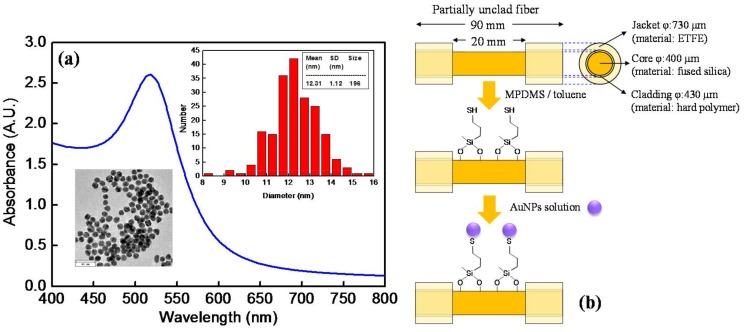
(**a**) Absorption spectrum of AuNP solution. Insets: TEM image of AuNPs and size distribution of the AuNPs in the image; (**b**) Schematic of modification of the partially unclad fiber with AuNPs.

Gold nanoparticles (AuNPs) were synthesized following a previously reported procedure [5,52]. An aqueous solution of HAuCl_4_ solution (0.88 mM, 50 mL) was heated to boiling with vigorous stirring for about 15 min. Then a freshly prepared trisodium citrate solution (1%, 6 mL) was rapidly added in the boiling solution. The solution was kept boiling for 10 min and the color changed from yellow to colorless, then black purple, and finally to ruby-red. After the solution turned to ruby-red color, it was kept boiling with stirring for 10 min, then it was allowed to cool down to room temperature and stored as a stock solution. A representative UV-vis spectrum of the AuNP solution, which was obtained by a double beam UV-Visible spectrometer (Cintra 202, GBC, Dandenong, VIC, Australia), is shown in Figure 2a. The peak wavelength and the peak absorbance of the AuNP solution was around 518 nm and 2.5 A.U., respectively. The image and histogram of particle diameter of the AuNPs, as shown in the Inset of Figure 2a, were obtained by a transmission election microscope (TEM, 1200 EX, JEOL, Tokyo, Japan). By TEM image analysis, the average diameter of the AuNPs is 12.3 ± 1.1 nm. To prepare sensing fibers with four different surface coverages of AuNPs, the stock AuNP solution was diluted to four different concentrations with the peak absorbance at 518 nm to be 0.5 A.U, 1.0 A.U, 1.5 A.U, and 2.0 A.U.

The optical fibers used as the sensing fibers were multimode hard clad silica fiber (model: 0.37 NA Low OH Optical Fiber, Item # CF01493-12, OFS, Norcross, GA, USA) with core, cladding and coating diameters (*φ*) of 400, 430, and 730 μm, respectively. The core was made of fused silica material, the cladding was made of hard polymer, and the coating was made of ethylene tetrafluoroethylene (ETFE) material. A section of 20 mm of the coating and cladding in the middle section of the optical fibers (total length = 90 mm) was removed by using a CO_2_ laser processing system (V-460, Universal Laser Systems Inc., Scottsdale, AZ, USA) to form a sensing window. The choice of the length of the unclad fiber is based on an optimization study as reported previously [23]. The fabrication quality analysis for the laser-processed optical fiber sensors follows a method as previously reported [38]. The quality assurance method provides superior quality verification of the materials properties of the optical fiber sensors to eliminate any concerns regarding the fiber damage in the unclad region and the subsequent change of the refractive index in that area. Then the fiber end faces were polished to an optically smooth surface to increase the transmission efficiency. Such partially unclad fibers after modification with AuNPs were used as the sensing fibers [52]. Before modification with AuNPs on the sensing window, the partially unclad fibers were cleaned by a mixture of H_2_SO_4_ and H_2_O_2_ with volume ratio of 3:2. After cleaning, the partially unclad fibers were immersed in a solution of (3-mercaptopropyl)methyldimethoxysilane (MPDMS) in toluene (volume ratio = 1:49) for 4 h to functionalize the sensing window with thiol group. The MPDMS solution was prehydrolyzed overnight before use. After rinsing, four sets of the partially unclad fibers were separately immersed in AuNP solutions of four different concentrations for 2 h to allow the AuNPs to self-assemble on the sensing window of the partially unclad fibers as shown in Figure 2b.

#### 2.2.2. Fiber Optic Extinction Spectrum Measurement System

**Figure 3:**
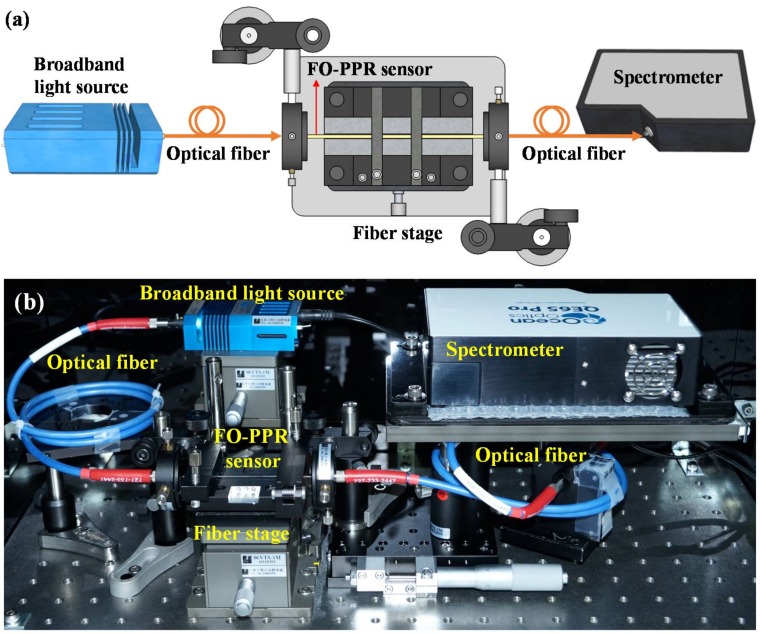
Setup of the fiber optic extinction spectrum measurement system: (**a**) schematic and (**b**) illustration.

In order to verify the theoretical model for extinction spectrum of AuNPs on the partially unclad fibers under evanescent-wave excitation, a fiber optic extinction spectrum measurement system was established, as shown in Figure 3. The system was consisted of a broadband light source of wavelength range from 200 nm to 2000 nm (model: LS-1, Ocean Optics Inc., Winter Park, FL, USA), an optical fiber to guide the incident light into the proximal end of a sensing fiber, a fiber holder to house the sensing fiber, an optical fiber to collect the light exiting the distal end of the sensing fiber for detection, and a spectrometer (QE-65 pro, Ocean Optics Inc.). The fiber holder was also designed to accommodate a microfluidic chip which holds the sensing fiber to measure the sensor responses under solutions of different refractive indexes [53].

## 3. Results and Discussion

### 3.1. Extinction Spectrum

**Figure 4:**
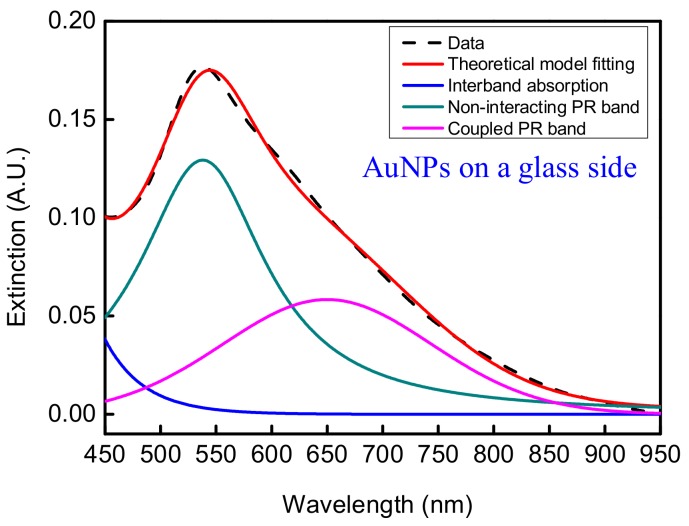
Extinction spectrum of AuNPs on a glass slide and the deconvoluted bands.

In order to verify the theoretical model, we immobilize the AuNPs on the fiber core and glass slide surfaces. The UV-vis spectrum of the AuNPs on a glass slide, as shown by the black dash line of Figure 4, was obtained by a double beam UV-Visible spectrometer (Cintra 202, GBC). Then the least square method (Levenberg-Marquardt optimization scheme) was used to fit the spectral data, as shown by the red line of Figure 4 (coefficient of determination R^2^ = 0.9983), and to deconvolute the overall extinction spectrum to a combination of bands (interband absorption, the non-interacting PR band, and the coupled PR band) as shown by the blue, green, and pink lines in Figure 4, respectively. After deconvolution, the peak wavelengths of the non-interacting PR band and the coupled PR band are 538 nm and 645 nm, respectively, while the full-width at half-maximum (FWHM) of the non-interacting PR band *w* is 149 nm, and the FWHM of the coupled PR band *w*_2*rd*_ is 229 nm.

**Figure 5:**
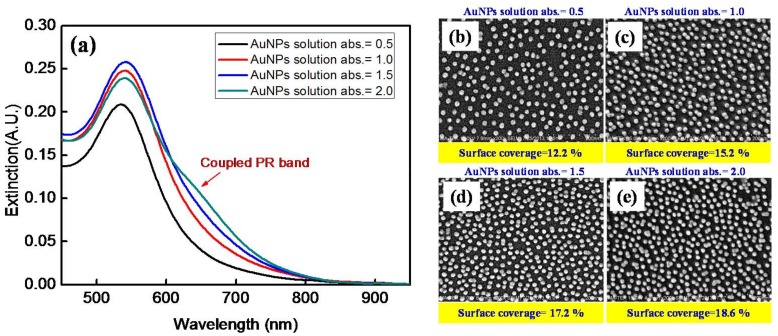
AuNPs on the fiber core surfaces prepared by immersion in AuNP solutions having peak absorbance, *A_λ_*, at 518 nm of 0.5, 1.0, 1.5, and 2.0: (**a**) Extinction spectra and (**b**–**e**) SEM images with *A_λ_* at (**b**) 0.5; (**c**) 1.0; (**d**) 1.5; (**e**) 2.0.

**Figure 6:**
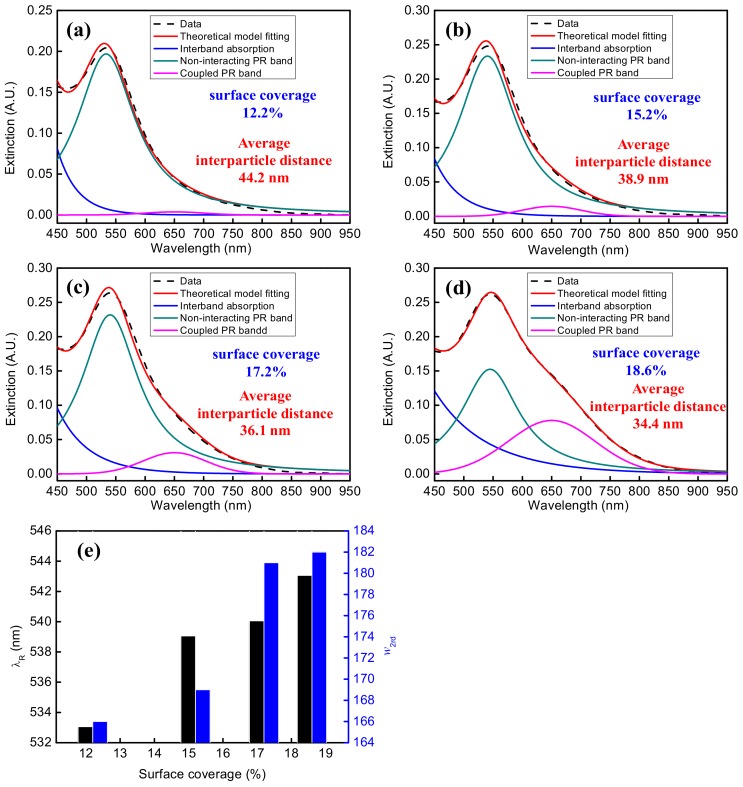
Experimental, simulated, and deconvoluted extinction spectra of AuNPs on the fiber core surfaces prepared by immersion in AuNP solutions having peak absorbance, *A_λ_*, at 518 nm of (**a**) 0.5; (**b**) 1.0; (**c**) 1.5; and (**d**) 2.0; (**e**) relationships between *λ_R_* and *w*_2*rd*_ versus surface coverage.

The extinction spectra of AuNPs on the fiber core surfaces prepared by immersion in the AuNP solutions having the peak absorbance at 518 nm of 0.5, 1.0, 1.5, and 2.0, are shown in Figure 5a. The SEM images of the four fiber core surfaces are shown in Figure 5b–e, respectively. According to the image analysis method [54], the respective surface particle densities are 1027 particles/μm^2^, 1272 particles/μm^2^, 1444 particles/μm^2^, and 1557 particles/μm^2^, which correspond to the surface coverages of 12.2%, 15.2%, 17.2%, and 18.6%, respectively, while the average center-to-center interparticle distances are 44.2 nm, 38.9 nm, 36.1 nm, 34.4 nm, respectively. As shown in Figure 6 and Table 1, the peak wavelength of the non-interacting PR band shifts, *λ_R_*, to longer wavelength with increasing surface coverage of the AuNPs on the fiber core surface. It had been found that the shift drops to zero when the gap between two AuNPs reaches about 2.5 times the particle size [47,48], suggesting that plasmon coupling is insignificant when the interparticle distance is more than about 3.5 times the particle size, which is about 43 nm in this study. Therefore, the average interparticle distances in the above four fiber core surfaces except the one with lowest surface coverage should still lead to weak plasmon coupling, and hence, red-shifts of the non-interacting PR band with increasing surface coverage. However, it should be noted that the FWHM of the non-interacting PR band, *w*_2*rd*_, is essentially constant when the surface coverage varies. Furthermore, examining the effect of surface coverage on the coupled PR band, it is found that the peak wavelength remains at 645 nm but the FWHM increases with higher surface coverage.

To characterize the distribution of the AuNPs on the fiber core surfaces, we define a term absorbance ratio, AR, as the peak absorbance of the coupled PR band of AuNPs to the peak absorbance of the non-interacting PR band. By this definition, ARs of the four extinction spectra are 0.004:0.20 = 0.020, 0.015:0.23 = 0.065, 0.031:0.23 = 0.13, and 0.082:0.19 = 0.43, respectively. When the surface coverage is 12.2%, the effect of plasmon coupling band can almost be ignored. When the surface coverage increases, the absorbance of the non-interacting PR band gradually decreases, while the absorbance of the coupled PR band gradually increases. These results agree with the behavior of coupled noble metal nanoparticles as reported in literature [47,55,56].

### 3.2. FOPPR Refractive Index Sensing

**Figure 7:**
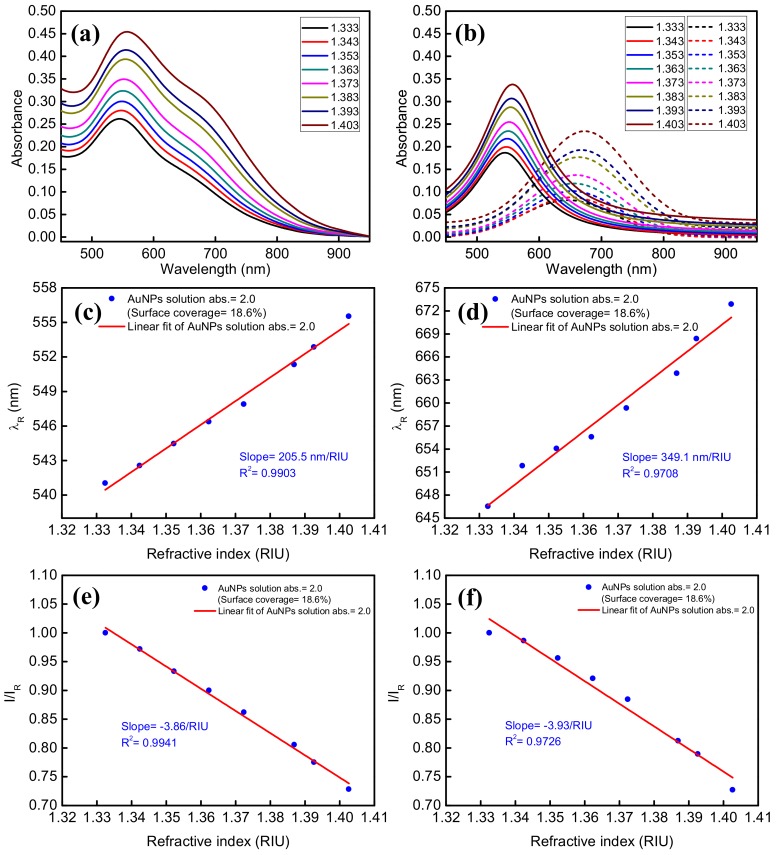
(**a**) Experimental and (**b**) deconvoluted extinction spectra of AuNPs on the fiber core surfaces under solutions of different RIs (solid line: non-interacting PR band, dashed line: coupled PR band); (**c**) plot of *λ_R_* verus RI for the non-interacting PR band; (**d**) plot of *λ_R_* verus RI for the coupled PR band; (**e**) plot of normalized intensity vs RI for the non-interacting PR band; (**f**) plot of normalized intensity vs. RI for the coupled PR band. Preparation condition: immersion in an AuNP solution having peak absorbance, *A_λ_*, at 518 nm of 2.0.

To investigate the sensor sensitivity of the two kinds of PR bands, the non-interacting PR band and the coupled PR band, a sensing fiber prepared in an AuNP solution of absorbance = 2.0 was encapsulated into a microfluidic chip. During the experiments, the air conditioner was turned on and the temperature was set at 25 degrees Celsius. Eight different concentrations of sucrose solution [57] from low refractive index (RI) into high RI were injected into the microfluidic chip serially under ambient conditions, and the extinction spectra at different refractive indices as shown in Figure 7a were measured using the fiber optic extinction spectrum measurement system. The RIs corresponding to the eight different concentrations were measured with an Abbe refractometer (model: RA-620, KEM Inc., Tokyo, Japan), and the results were 1.3325, 1.3424, 1.3523 1.3623, 1.3724, 1.3869, 1.3926, and 1.4027. The deconvoluted extinction spectra according to the theoretical model in Section 2.1 are shown in Figure 7b. The peak wavelength and peak absorbance of the deconvoluted PR bands were then used to estimate the wavelength sensitivity and the extinction sensitivity, which are defined by the slopes of the plots of peak wavelength and peak absorbance versus RI of the solution, respectively. The results are shown in Figure 7c,d (the red curve is the fitting curve). Both the peak wavelength (*λ_R_*) of the non-interacting PR band and the peak wavelength (*λ*_2*rd*_) of the coupled PR band increase with the increase of the RI of the solution. The wavelength sensitivities are 205.5 nm/RIU and 349.1 nm/RIU, respectively. These values are significantly larger than those measured by transmission through an AuNP sub-monolayer on a slide [22,58] or in solution [59] or by theoretical simulations [32], and is consistent with a previous report [29]. It should be noted that the wavelength sensitivity has been found to be size dependent and has higher value at larger size [58] and thus the comparison here is based on AuNPs of a similar size. The higher wavelength sensitivity by FOPPR interrogation may be attributed to the excitation of transverse-electric propagating guided modes in the monolayer of sparse and disordered AuNPs, characterized by extremely high levels of light absorption [31]. Once the waveguiding behavior set in, more than 3-fold amplification of wavelength sensitivity with respect to isolated AuNPs had been reported [31]. In addition, the wavelength sensitivity of the coupled PR band is higher than that of the non-interacting PR band, which may be attributed to the higher RI sensitivity of anisotropic gold nanostructures [59,60] and the near-field interaction of the coupled-particle system [50,58].

In terms of extinction sensitivity, we define the light intensity at 530 nm when the AuNPs on fiber core surface is under pure water (RI = 1.333) as *I_R_*, the light intensity at the peak wavelength under other RI is *I*, and the sensor response is taken as the normalized intensity (*I*/*I_R_*). Then the normalized intensity is plotted against the RI of the solution. As shown in Figure 7e,f (the red line is the fitting curve), both the normalized intensities of the deconvoluted non-interacting PR and coupled PR bands decrease with the increase of RI, and the extinction sensitivities are −3.86/RIU and −3.93/RIU, respectively.

**Figure 8:**
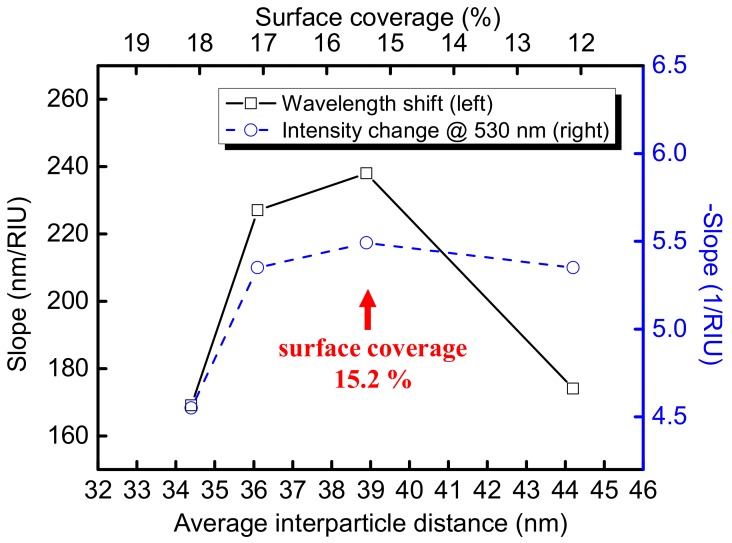
Plots of wavelength sensitivity and extinction sensitivity versus average interparticle distance.

To investigate the effect of surface coverage of AuNPs on the fiber core to both the wavelength sensitivity and extinction sensitivity, four different surface coverages (i.e., different average interparticle distances) were selected for the analysis of the surface coverage effect to the band at about 530 nm without deconvolution, since the band at about 530 nm directly related to the FOPPR sensor response experimentally. As shown in Figure 8, maxima appear at a surface coverage of 15.2% for both plots. On the contrary, previous studies either show higher wavelength sensitivity [34] and extinction sensitivity [61] at increasing surface coverage or lower wavelength sensitivity at increasing surface coverage [33]. It should be noted that previous studies used nanoparticle sizes larger than that in this study and also used different ranges of surface coverages. Our approach enables the exploitation of the effect of surface coverage on both wavelength sensitivity and extinction sensitivity and also allow us to understand the effect of surface coverage to both the non-interacting PR and coupled PR bands. Thus, a suitable surface coverage of AuNPs with a matched excitation wavelength in a FOPPR sensor could be optimized by this approach.

## 4. Conclusions

In this study, a fiber-optic extinction spectrum measurement system was successfully established, and a theoretical model was used to analyze the coverage of AuNPs on the fiber core surface. This theoretical model can be used to analyze the plasmon resonance band of AuNPs on the core surface of an optical fiber, such as interband absorption of AuNPs, non-interacting PR band due to isolated AuNPs, and coupled PR band of interacting AuNPs. By this approach, the effect of surface coverage of AuNPS on the fiber core surface to the plasmon resonance band can be systematically explored. When the surface coverage is smaller than 12.2%, the plasmon coupling effect can be almost ignored.

This method is also applied to the examination of the refractive index sensitivity of the FOPPR sensor with respect to the non-interacting PR band and the coupled PR band. In terms of wavelength sensitivity, the refractive index sensitivity of the coupled PR band is greater than that of the non-interacting PR band. The two wavelength sensitivities obtained are 205.5 nm/RIU and 349.1 nm/RIU, respectively. In terms of extinction sensitivity, refractive index sensitivity of the coupled PR band is similar to that of the non-interacting PR band at a surface coverage of 18.6%. The two extinction sensitivities obtained are −3.86/RIU and −3.93/RIU, respectively.

Through this fiber-optic extinction spectrometry system, it is possible to understand the degree of surface coverage of the AuNPs on the fiber core surface and hence to optimize the refractive index sensitivity of the FOPPR sensor. Besides the study of bulk the refractive index sensitivity in this work, the effect of plasmon coupling to the biosensor sensitivity will be explored in our future work. This will help to develop the FOPPR sensor with high sensor sensitivity and sensor resolution.

## Figures and Tables

**Table 1:** Surface coverage, average interparticle distance, and experimental values of peak wavelengths, peak absorbances, and FWHMs extracted from the FO-PPR extinction spectra.

	Percent Surface Coverage (%)	Average Interparticle Distance (nm)	Non-Interacting PR Band		Coupled PR Band		*w* (nm)	*w*_2*rd*_ (nm)	*R*^2^ Coefficient of Determination
			*λ_R_* (nm)	*A_λ_* (A.U.)	*λ_2rd_* (nm)	*A_λ_* (A.U.)			
AuNP solution with *A_λ_* = 0.5	12.2	44.2	533	0.2	645	0.004	135	166	0.9967
AuNP solution with *A_λ_* = 1.0	15.2	38.9	539	0.23	645	0.015	136	169	0.9969
AuNP solution with *A_λ_* = 1.5	17.2	36.1	540	0.23	645	0.031	135	181	0.9970
AuNP solution with *A_λ_* = 2.0	18.6	34.4	543	0.19	645	0.082	132	182	0.9996
